# Effect of PVP-Capped ZnO Nanoparticles with Enhanced Charge Transport on the Performance of P3HT/PCBM Polymer Solar Cells

**DOI:** 10.3390/polym11111818

**Published:** 2019-11-05

**Authors:** OkSik Kim, JinBeom Kwon, SaeWan Kim, Binrui Xu, KyeongHo Seo, CheolEon Park, WooJong Do, JinHyuk Bae, ShinWon Kang

**Affiliations:** 1 School of Electronics Engineering, College of IT Engineering, Kyungpook National University, 1370 Sankyuk-dong, Daegu 702-701, Korea; oskim@knu.ac.kr (O.K.); jinbumkwon@naver.com (J.K.); kei95304@gmail.com (S.K.); kezherui123@gmail.com (B.X.); tjrudgh0826@naver.com (K.S.); jhbae@ee.knu.ac.kr (J.B.); 2 Center for Robotics Research, Korea Institute of Science and Technology (KIST), 5 Hwarang-ro 14-gil, Seongbuk-gu, Seoul 02792, Korea; skcjfdjs@naver.com; 3 Department of Sensor and Display Engineering, Kyungpook National University, 1370 Sankyuk-dong, Daegu 702-701, Korea; loseo@knu.ac.kr

**Keywords:** polymer solar cells, bulk-heterojunction, ZnO, surface modulation, polyvinyl pyrrolidone, PVP, oxygen

## Abstract

We attempted surface modification in ZnO nanoparticles (NPs) synthesized by the sol–gel process with polyvinyl pyrrolidone (PVP) applied to bulk-heterojunction polymer solar cells (PSCs) as an electron transport layer (ETL). In general, ZnO NPs have trap sites due to oxygen vacancies which capture electrons and degrade the performance of the PSCs. Devices with six different PVP:Zn ratios (0.615 g, 1.230 g, 1.846 g, 2.460 g, 3.075 g, and 3.690 g) were fabricated for surface modification, and the optimized PVP:Zn ratio (2.460 g) was found for PSCs based on P3HT/PCBM. The power conversion efficiency (PCE) of the fabricated PSCs with PVP-capped ZnO exhibited a significant increase of approximately 21% in PCE and excellent air-stability as compared with the uncapped ZnO-based PSCs.

## 1. Introduction

Recently, bulk-heterojunction (BHJ) polymer solar cells (PSCs) have received considerable attention due to their many merits, such as light weight, low-cost manufacture, flexibility, roll-to-roll fabrication process compatibility, and bandgap tenability [1,2,3]. Since the first report on PSCs in 1986, various studies have been conducted to improve their performance [4]. Significant efforts have been made to improve the power conversion efficiency (PCE) through unconventional donor–acceptor materials [5,6,7,8], advanced device architectures such as inverted structures of PSCs [9], new additives and dopant materials [10,11,12], optimized interfaces [13], etc. In particular, inverted structures of PSCs have been studied because of their superior device stability for high PCE [9]. In both conventional and inverted devices, to overcome the drawbacks of fast degradation with acidic PEDOT:PSS and the metal oxide layer [14], the active material simply performs the role of absorbing photons and converting them into free charge carriers. However, to achieve good performance, a hole transport layer (HTL) and electron transport layer (ETL) are required to improve the excellent transportability of electron/hole carriers [15,16,17,18,19,20,21]. Litzov et al. reported that an excellent material should fulfill the conditions for the ETL and HTL, which require transparency, good electrical properties, and chemical stability [22]. Baglio et al. reported a dye-sensitized solar cell with titanium oxide (TiO_x_) as the ETL [23]. Hadipour et al. presented a BHJ device using TiO_x_ at room temperature with the solution process as the ETL [24]. Li et al. reported PSCs using Cs_2_CO_3_ in combination with V_2_O_5_ as the HTL [9]. Wiranwetchayan et al. reported inverted-structure PSCs using Nb_2_O_5_ as the ETL [25]. In particular, ZnO NPs are used for various optoelectronic devices due to their excellent environmental stability and electron mobility [26]. The ZnO thin films have been grown by different techniques, such as chemical vapor deposition, sputtering, sol–gel, pulsed laser deposition, and atomic layer deposition (ALD). Recently, ALD is the preferred technology due to the possibility of a low-temperature process [27]. Jan Gilot et al. demonstrated a PSC with ZnO as an optical spacer where the short-circuit current density (*J*_sc_) increased as a result of increased light absorption and redistribution of the electric field inside the active layer [28]. These various attempts achieved significant improvements in results and achieved a PCE in excess of 11% [29]. Recently, many studies have been conducted on the synthesis, growth, and defect engineering of ZnO nanoparticles (NPs), especially those with oxygen vacancies [30,31,32]. Among them, researchers have conducted studies on the surface modification of ZnO NPs with various capping agents to reduce defects through encapsulation of the surface [33,34]. In particular, we attempted surface modification in ZnO NPs with polyvinyl pyrrolidone (PVP) [35,36,37] applied to PSCs as an ETL; surface modification improved the trap-induced defects due to oxygen defects on the surface, thereby preventing the trapping of electrons and reduction of performance. Devices with six different PVP:Zn ratios (0.615 g, 1.230 g, 1.846 g, 2.460 g, 3.075 g, and 3.690 g) were fabricated, and the optimized PVP:Zn ratio (2.460 g) was found for PSCs based on P3HT/PCBM. The PCE of the fabricated PSCs with PVP-capped ZnO exhibited a significant increase of approximately 21% in PCE compared with the uncapped ZnO-based PSCs.

Therefore, it has been confirmed that it is very important to reduce trap-related states due to oxygen deficiency for efficient electron transportation.

## 2. Experimental

**Figure 1:**
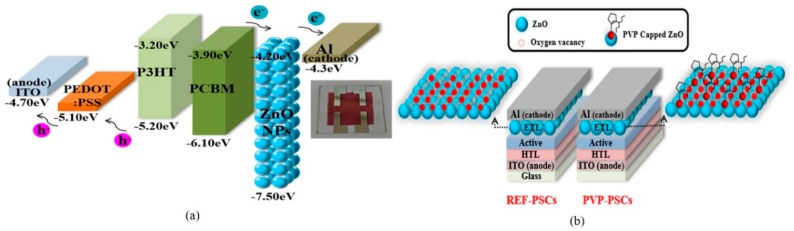
Schematic diagram of fabricated bulk-heterojunction (BHJ) polymer solar cells (PSCs). (**a**) Energy band diagram. (**b**) Structure and films state of uncapped-ZnO nanoparticles (NPs) and polyvinyl pyrrolidone (PVP)-capped ZnO NPs.

The PSC device structure consists of an anode (ITO), an HTL, an active layer (P3HT:PCBM, Luminescence Technology Corp., Hsin Chu, Taiwan), an ETL (PVP (Sigma-Aldrich, Saint Louis, MO, USA)-capped ZnO), and a cathode (aluminum), as shown Figure 1. The patterned ITO glasses were cleaned with acetone, methanol, and isopropyl alcohol for 10 min in an ultrasonic bath, followed by UV–ozone curing for 15 min. The HTL was formed using PEDOT:PSS (poly(ethylenedioxythiophene):polystyrene sulfonate, Baytron P AI 4083, H. C. Starck, Newton, MA, USA) on patterned ITO with spin-coating and thermal annealing treatment at 150 °C for 10 min. Thereafter, the active layer was formed using the mixing of the blend components where P3HT and PCBM (1:1 wt %) were dissolved in 1,2-Dichlorobenzene (Sigma-Aldrich). Subsequently, the PVP-capped ZnO NP solution was coated to form the ETL and thermally annealed under vacuum conditions. Finally, a top cathode of Al was evaporated at a pressure of 10^−6^ Torr. The active area of the fabricated PSCs was 9 mm^2^ (defined by the shadow mask of the vertical overlap of ITO and Al, electrodes). A modified and optimized sol–gel method was used for the synthesis of ZnO NPs. A total of 2.46 g Zinc acetate dihydrate (Sigma-Aldrich) was dissolved in 110 mL of methanol, and 0.96 g of potassium hydroxide was dissolved in 50 mL of methanol. PVP (Sigma-Aldrich) was dissolved in methanol. The KOH solution and PVP solution were added dropwise into a 200 mL flask filled with the Zn(AC)_2_·2H_2_O solution and stirred at 60 °C for 1 hour. Then, isopropanol and hexane were added to the mixture overnight. The PVP-capped ZnO NPs were gathered by centrifuging and washed twice to purify the prepared PVP-capped ZnO and re-dispersed in ethanol (10 mg/mL). The schematic of the device structure and band diagram are shown in Figure 1. The PV performance measurements were performed on Agilent 4155C semiconductor parameter (Agilent, Santa Clara, CA, USA) analyzer integrated with a Newport xenon lamp as a solar simulator (AM 1.5). A National Renewable Energy Laboratory (NREL) calibrated silicon photodetector was used to calibrate the light source with a light intensity of 100 mW·cm^−2^.

Photoluminescence (PL, QE65000, Ocean Optics, Largo, FL, USA) characteristics were assessed by a spectrum analyzer to measure the excitation and bandgap of PVP-capped ZnO NPs. To verify the coordination of ZnO with PVP molecules, Fourier transform infrared (FT-IR) spectra were obtained from FT-IR spectroscopy (Frontier, PerkinElmer, Waltham, MA, USA). UV-visible absorption characteristics were examined with a UV-visible spectrophotometer (UV-1601, Shimadzu Corp., Kyoto, Japan). External quantum efficiency (EQE) spectra were measured using a measuring kit (Quantx-300 system, Newport, Irvine, CA, USA). Field emission-scanning electron microscopy (FE-SEM, SU8220, Hitachi, Tokyo, Japan) was used to evaluate the thickness of the cross-sectional image of BHJ-PSCs with uncapped ZnO NPs and PVP-capped ZnO NPs. The morphological modifications, such as two-dimensional (2D) and three-dimensional (3D) atomic force microscopy (AFM) images of the surface of uncapped ZnO NPs and PVP-capped ZnO NPs as the ETL, were obtained with an AFM microscope (5500 Agilent Technologies, Santa Clara, CA, USA).

## 3. Results and Discussion

**Figure 2:**
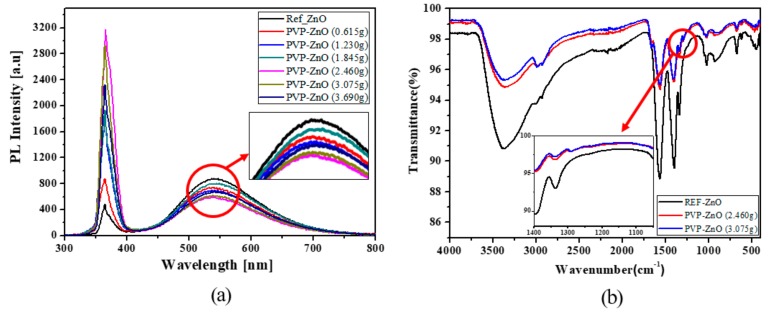
(**a**) Photoluminescence (PL) spectra of PVP-ZnO NPs (0.615 g, 1.230 g, 1.845 g, 2.460 g, 3.075 g, and 3.690 g) and Ref-ZnO, (**b**) FT-IR spectra of PVP-ZnO NPs (2.460 g and 3.075 g) and Ref-ZnO.

Figure 2a shows the photoluminescence (PL) of uncapped ZnO (Ref-ZnO) NPs and PVP-capped ZnO (PVP-ZnO) NPs with various PVP:Zn ratios. The PL peak near 550 nm is the visible light emission from the trap induced by oxygen deficiency at the surface of the ZnO NPs, and the PL peak near 370 nm is the radiation from the ZnO NP direct bandgap. The UV radiations increased dramatically by approximately 6.5 times, from 0.615 to 2.460 g, from PVP-ZnO NPs, and visible light emissions were weakened. However, in Ref-ZnO NPs, the UV radiation was extremely weak and the visible light emission was somewhat strong. Interestingly, the UV radiations decreased slightly beyond 2.460 g of PVP-ZnO NPs, from 3.075 to 3.690 g of PVP-ZnO NPs, and visible light emissions were also weaker than 2.460 g of the PVP-ZnO NPs [35]. These results show that the visible light emission was due to the deep trap state at the surface of the ZnO NPs. Consequently, when capped with the proper ratios of PVP:Zn, the deep trap state can be eliminated. Figure 2b shows the Fourier transform infrared spectroscopy (FT-IR) of Ref-ZnO NPs and PVP-ZnO NPs (2.460 g and 3.075 g, respectively). Through the FT-IR results of Ref-ZnO, we can observe the peaks by number located at 3372, 1567, and 451 cm^−1^ are assigned to the O–H stretching vibration, symmetric C=O stretching of zinc acetate dehydrate, and ZnO stretching vibrations, respectively. In the spectrum of PVP-ZnO, the band at 1655 cm^−1^ is assigned to the stretching vibration of the C=O in the PVP. We can observe a peak at 1288 cm^−1^ for PVP-ZnO NPs, which is believed to be due to the chemical reaction between PVP and ZnO NPs [36].

**Figure 3:**
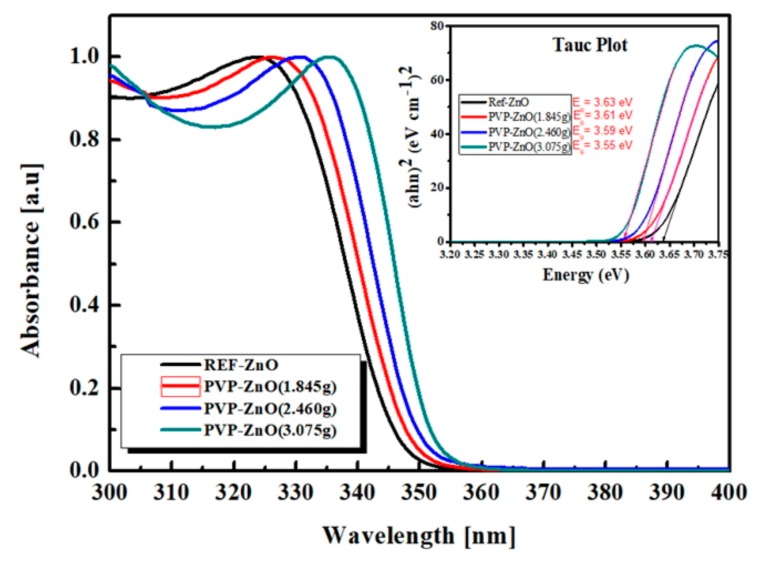
UV-visible absorption spectra of PVP-ZnO NPs (1.845 g, 2.460 g, 3.075 g) and Ref-ZnO NPs. Insert showing bandgap energy using Tauc plot from UV-visible absorption spectra.

Figure 3 shows the UV-visible absorption spectra of Ref-ZnO NPs and PVP-ZnO NPs with three different PVP:Zn ratios (1.845 g, 2.460 g, and 3.075 g). To confirm the size of particles according to surface modification, the average particle size of nanoparticles was estimated using the effective mass approximation formula [38,39] proposed by Meulenkamp [31] as follows:

$$\frac{1240}{\lambda_{1/2}}=a+\;\frac{b}{D^{2}}+\frac{c}{D}$$

where λ_1/2_ = the wavelength of 1/2 the value of the maximum peak (nm), *D* = the radius of particle (Å), and *a*, *b*, and *c* = the constants of 3.556, 799.9, and 22.64, respectively. The particle size values obtained from the formula proposed by Meulenkamp for the three different PVP:Zn ratios are listed in Table 1. The particle size values are 3.06, 3.15, 3.23, and 3.39 nm for Ref-ZnO, PVP-ZnO (1.845 g), PVP-ZnO (2.460 g), and PVP-ZnO (3.075 g), respectively. Using the Tauc plot, the values of optical bandgap energy from the absorption data are listed in Figure 3 and Table 1. It was observed that as we increased the capping agent concentration, the particle size increased and the bandgap reduced [40]. The size distribution of individual particles ranged from 3.06 to 3.39 nm, and particle sizes increased slightly upon PVP surface modification. The red shifts of UV-visible absorption peaks occurred in response to the increased aging time accompanying the growth of ZnO NPs.

**Figure 4:**
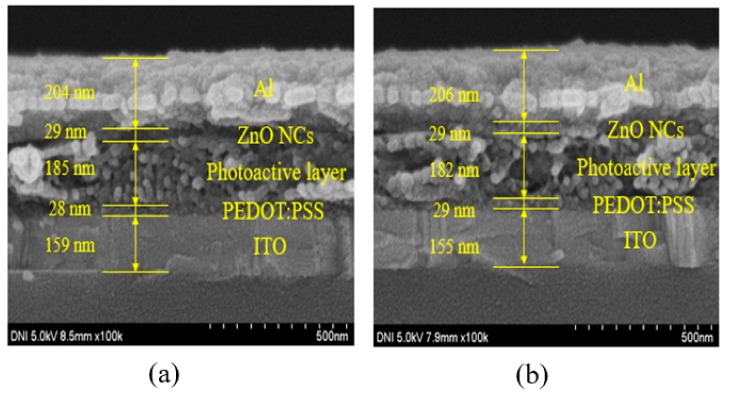
FE-SEM cross-sectional images of (**a**) BHJ-PSCs fabricated with bare ZnO NPs (**b**) and BHJ-PSCs fabricated with PVP-capped ZnO NPs.

Figure 4a shows the cross-sectional image of the BHJ-PSCs fabricated with Ref-ZnO NPs. The thickness of the Ref-ZnO thin films was estimated to be 28–30 nm. Figure 4b shows the cross-sectional image of the BHJ-PSCs fabricated with PVP-ZnO NPs. The thickness of the PVP-ZnO thin films was estimated to be 28–30 nm. The ZnO layer thicknesses of the two samples were similar regardless of PVP capping.

**Figure 5:**
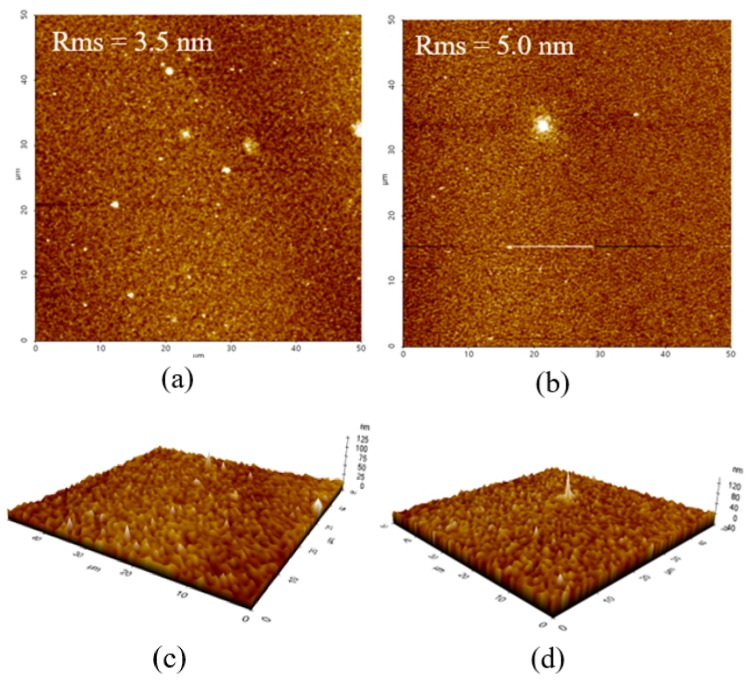
Atomic force microscopy (AFM) images of Ref-ZnO NPs and PVP-ZnO NPs on top of P3HT/PCBM photoactive layers: (**a**) Ref-ZnO film, (**b**) PVP-ZnO film. 3D AFM images of Ref-ZnO NPs and PVP-ZnO NPs on top of P3HT/PCBM photoactive layers: (**c**) Ref-ZnO film, (**d**) PVP-ZnO film.

To investigate how the inclusion of PVP-ZnO NPs influences the ETL morphology, the surface morphology was examined by AFM, and the results are shown in Figure 5. The Ref-ZnO thin film shows a root mean square (RMS) roughness value of 3.5 nm, and the PVP-ZnO thin film shows a slightly higher RMS roughness value of 5.0 nm. The Ref-ZnO thin films on the active layer exhibit a flat island morphology with a height of 42.1 nm and a diameter of 1.25 µm. In the case of PVP-ZnO thin films on the active layer, the island shape was scattered with a height of 63.3 nm and a diameter of 1.36 µm. This shows that ZnO formation is dispersed and provides an additional path for electron transfer. When aluminum was deposited on top of the small, swollen island of the surface, the contact area increased to promote efficient electron transfer to the metal electrode.

**Figure 6:**
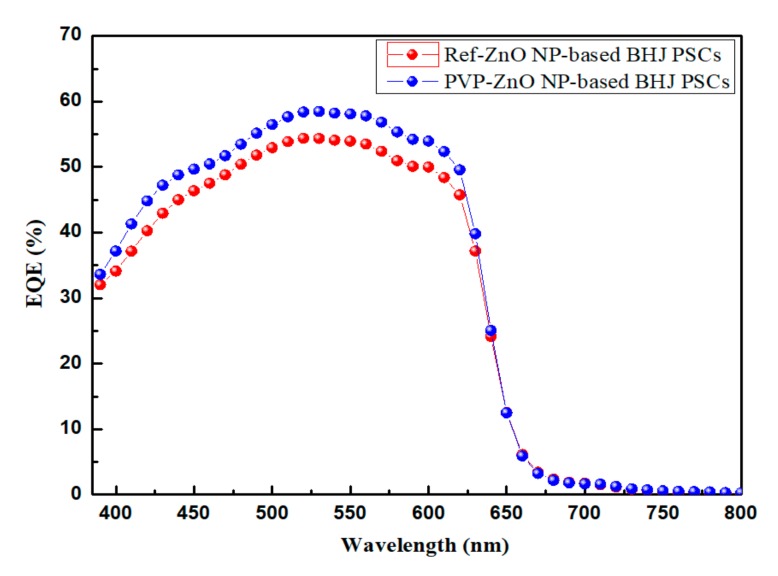
External quantum efficiency characteristics of (**Red**) Ref-ZnO NP-based BHJ PSCs and (**Blue**) PVP-ZnO NP-based BHJ PSCs.

In Table 2, the BHJ PSCs with uncapped ZnO NPs (Ref-PSCs) fabricated as a reference exhibited an open-circuit voltage (*V*_oc_) of 0.593 V, a short-circuit current density (*J*_sc_) of 6.327 mA/cm^2^, a fill factor (FF) of 0.645, and a maximum PCE of 2.49%. In contrast, in the best BHJ PSCs with PVP-capped ZnO NPs (PVP-PSCs), *J*_sc_ and PCE were significantly increased by more 17% and 21%, respectively. The improved PCE is mainly attributed to the improved *J*_sc_. In Figure 6, the increase of *J*_sc_ is consistent with EQE spectra. The EQE spectrum obtained for the PVP-PSCs is broad over a wide range (360~630 nm). The reason for this is that, in the synthesis of ZnO NPs, the oxygen vacancy defect present on the surface of ZnO NPs captured electrons. This caused charge imbalance, which impeded efficient charge transport in the PSCs. However, when PVP is capped by electrostatic interactions on the surface of ZnO NPs, inner defects and surface traps can be reduced [41]. Additionally, an increase in concentration of more than 2.460 g of PVP adversely affects the photovoltaic performance of the device due to the *J*_sc_ decrease. Relatively moderate PVP particle concentration in the range of 2.460~3.075 g was found to significantly enhance the *J*_sc_ of BHJ PSCs based on PVP-ZnO NPs [42]. The electron mobilities of uncapped ZnO and PVP-capped ZnO films could be calculated through space charge limited current (SCLC) model, following the Mott–Gurney SCLC equation [43]:

$$J=\frac{9}{8}\mu \varepsilon_{0}\varepsilon_{r}\frac{V_{a}^{2}}{d^{3}}$$

where μ, ε_0_, ε_r_, *V*_a_, and d are the charge carrier mobility, the vacuum dielectric constant (ε_0_ = 8.85 × 10^−12^ F/m), the dielectric constant of P3HT:PCBM (ε_r_ = 3) [44], the applied voltage, and the thickness of the active layer (confirmed by SEM image, d = 185 nm), respectively. The measured electron mobility was 1.43·10^−4^ cm^2^/VS (uncapped ZnO), 4.94·10^−4^ cm^2^/VS (PVP-capped ZnO), and it was confirmed that the electron mobility was 3.5 times improved by decreasing oxygen vacancy due to PVP-capping.

Stability is very important for BHJ PSCs. To gain more insight into device stability, we examined the stability of BHJ PSCs based on PVP-ZnO NPs and Ref-ZnO NPs under dark ambient atmosphere and different oxygen and water conditions at room temperature (about 25 ± 3 °C) for 360 h. The results showed that the PCE of PVP-PSCs significantly improved by about 21%. Moreover, the stability of PVP-PSCs was considerably improved, more than that of Ref-PSCs, which was significantly degraded when exposed to air, and they retained only 53% of their initial PCE after 360 h. However, for PVP-capped ZnO NPs-based BHJ PSCs, the PCE decreased to about 74% of the initial values, manifesting the vast potential of ZnO surface modification using PVP molecules for stable, scalable solar cells. The use of PVP-ZnO NPs as the ETL for BHJ PSCs effectively suppressed the light soaking of the conventional PSCs through the passivation of surface-absorbed oxygen defects.

**Figure 7:**
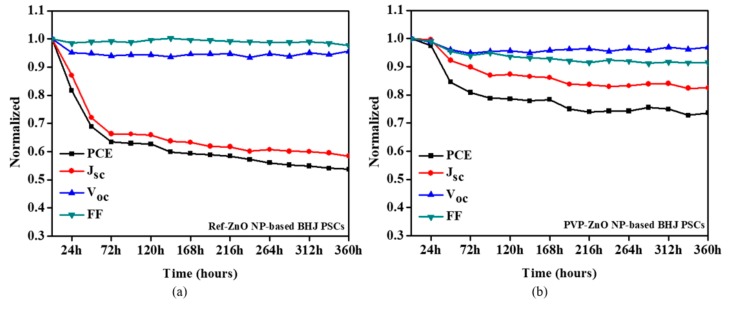
Normalized air-stability characteristics of (**a**) Ref-ZnO NP-based BHJ PSCs and (**b**) PVP-ZnO NP-based BHJ PSCs.

**Figure 8:**
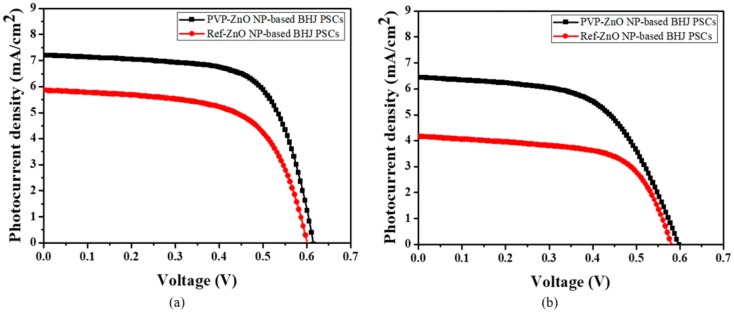
J–V characteristics of Ref-ZnO NP-based BHJ PSCs and PVP-ZnO NP-based BHJ PSCs at (**a**) 0 h and (**b**) 360 h.

In summary, PVP-ZnO NPs reduced oxygen vacancies from lattice defects and trapping sites, which allowed effective electron transfer in BHJ PSCs, resulting in an increase in *J*_sc_ and PCE. Moreover, as the surface traps and inner defects could be reduced, the device with PVP-PSCs exhibited excellent air stability and current density–voltage (J–V) curves after 360 h compared with the Ref-PSCs, as shown in Figure 7 and Figure 8.

## 4. Conclusions

In conclusion, in this work, we fabricated and appropriately characterized PVP-capped ZnO NPs as an effective ETL in PSCs fabricated to enhance charge balance and performance. It was shown that the PVP-capped ZnO NPs were very effective in reducing charge-trapping sites and oxygen vacancies from the cathode. The proposed BHJ PSCs with PVP-capped ZnO NPs as the ETL showed significantly enhanced PCE, current density, and air stability due to the reduced trap sites on the surface of ZnO NPs. Consequently, we confirmed that the BHJ PSC performance was improved due to effective electron transportation by reducing the defects of oxygen vacancies.

## Figures and Tables

**Table 1:** Average particle size and bandgap variation of ZnO nanoparticles with PVP:Zn ratios.

ETL	PVP Volume	Particle Size (nm)	Bandgap (*E*_g_) (eV)
REF-ZnO	-	3.06	3.63
PVP-ZnO (1.845g)	1.845g	3.15	3.61
PVP-ZnO (2.460g)	2.460g	3.23	3.59
PVP-ZnO (3.075g)	3.075g	3.39	3.55

**Table 2:** Photovoltaic performance parameters of BHJ-PSCs fabricated with ZnO and PVP-capped ZnO in different PVP:Zn ratios. PCE^a^ and PCE^b^ are best and average power conversion efficiencies (PCEs) of devices.

ETL	V_OC_ (V)	*J*_SC_ (mA/cm^2^)	*R*_S_ (ohm)	FF	PCE^a^ (%)	PCE^b^ (%)
REF-ZnO	0.593 ± 0.012	6.327 ± 0.002	139 ± 3.1	0.645 ± 0.034	2.49	2.46 ± 0.008
PVP-ZnO (0.615 g)	0.609 ± 0.004	6.840 ± 0.003	171 ± 2.9	0.580 ± 0.049	2.58	2.42 ± 0.024
PVP-ZnO (1.230 g)	0.616 ± 0.008	6.666 ± 0.004	159 ± 5.0	0.616 ± 0.056	2.59	2.53 ± 0.015
PVP-ZnO (1.845 g)	0.622 ± 0.004	6.132 ± 0.009	159 ± 8.1	0.629 ± 0.104	2.85	2.62 ± 0.073
PVP-ZnO (2.460 g)	0.617 ± 0.004	7.423 ± 0.004	138 ± 5.7	0.652 ± 0.072	3.08	2.98 ± 0.024
PVP-ZnO (3.075 g)	0.611 ± 0.004	7.277 ± 0.001	128 ± 1.2	0.662 ± 0.017	2.98	2.94 ± 0.008
PVP-ZnO (3.690 g)	0.614 ± 0.001	7.051 ± 0.003	164 ± 5.0	0.641 ± 0.044	2.85	2.78 ± 0.014
